# Interactions among filamentous fungi *Aspergillus niger*, *Fusarium verticillioides* and *Clonostachys rosea*: fungal biomass, diversity of secreted metabolites and fumonisin production

**DOI:** 10.1186/s12866-016-0698-3

**Published:** 2016-05-10

**Authors:** Subhankar Chatterjee, Yi Kuang, Richard Splivallo, Paramita Chatterjee, Petr Karlovsky

**Affiliations:** Molecular Phytopathology and Mycotoxin Research, Georg-August-University Göttingen, Grisebachstrasse 6, 37077 Göttingen, Germany

**Keywords:** Interference competition, Metabolic diversity, Fumonisin, Metabolic profiling

## Abstract

**Background:**

Interactions among fungi colonizing dead organic matter involve exploitation competition and interference competition. Major mechanism of interference competition is antibiosis caused by secreted secondary metabolites. The effect of competition on secondary metabolite production by fungi is however poorly understood. Fungal biomass was rarely monitored in interaction studies; it is not known whether dominance in pairwise interactions follows congruent patterns.

**Results:**

Pairwise interactions of three fungal species with different life styles were studied. The saprophyte *Aspergillus niger* (*A.n.*), the plant pathogen *Fusarium verticillioides* (*F.v.*), and the mycoparasite *Clonostachys rosea* (*C.r.*) were grown in single and dual cultures in minimal medium with asparagine as nitrogen source. Competitive fitness shifted with time: in dual *C.r.*/*F.v.* cultures after 10 d *F.v.* grew well while *C.r.* was suppressed*;* after 20 d *C.r.* recovered while *F.v.* became suppressed; and after 30 d most *F.v.* was destroyed. At certain time points fungal competitive fitness exhibited a rock–paper–scissors pattern: *F.v.* > *A.n.*, *A.n.* > *C.r.*, and *C.r.* > *F.v.* Most metabolites secreted to the medium at early stages in single and dual cultures were not found at later times. Many metabolites occurring in supernatants of single cultures were suppressed in dual cultures and many new metabolites not occurring in single cultures were found in dual cultures. *A. niger* showed the greatest ability to suppress the accumulation of metabolites produced by the other fungi. *A. niger* was also the species with the largest capacity of transforming metabolites produced by other fungi. Fumonisin production by *F. verticillioides* was suppressed in co-cultures with *C. rosea* but fumonisin B1 was not degraded by *C. rosea* nor did it affect the growth of *C. rosea* up to a concentration of 160 μg/ml.

**Conclusions:**

Competitive fitness in pairwise interactions among fungi is incongruent, indicating that species-specific factors and/or effects are involved. Many metabolites secreted by fungi are catabolized by their producers at later growth stages. Diversity of metabolites accumulating in the medium is stimulated by fungus/fungus interactions. *C. rosea* suppresses the synthesis of fumonisins by *F. verticillioides* but does not degrade fumonisins.

**Electronic supplementary material:**

The online version of this article (doi:10.1186/s12866-016-0698-3) contains supplementary material, which is available to authorized users.

## Background

Dead organic matter is rapidly colonized by a complex community of microorganisms that includes saprophytic fungi. Obligate saprophytic fungi feed on dead organic matter during their entire life, while many phytopathogenic and entomopathogenic fungi depend on saprophytic growth when they form propagules at the end of their life cycle. *Trichoderma*, *Gliocladium/Clonostachys*, and other mycoparasitic fungi with a wide range of hosts feed either on living fungal mycelia or on dead organic matter and may also colonize living plants. Interactions among saprophytic fungi are dominated by competition.

Interspecific competition is formally divided into exploitation competition, which occurs when the use of a resource by one species reduces its availability for another species, and interference competition, which occurs when one species directly restrains the growth or spread of a competitor. In microbial communities, exploitation competition reduces the access of a competitor to a substrate by its depletion while interference competition inhibits competitor's growth by antibiosis [1].

Filamentous fungi secrete cocktails of secondary metabolites, many of which possess prominent biological activities [2]. Although specific biological functions have been demonstrated for very few of them, these substances must contribute to the fitness of their producers at some stage of their life cycle or under specific, repeatedly occurring conditions. The ecological metabolite hypothesis postulates that secreted secondary metabolites modulate interactions of their producers with other organisms [3]. The current view of fungal ecological chemistry is that most, if not all, secreted fungal secondary metabolites are ecological metabolites [4]. Any physiological or developmental process may be the target of ecological metabolites. The best-established function of ecological metabolites is antibiotic activity that inhibits competing microorganisms [5]. Ecological metabolites can also inhibit the activity [6] or synthesis [7] of hydrolytic enzymes and the synthesis of other secondary metabolites [8, 9]. In response to antibiotic metabolites produced by a competitor, a fungus may detoxify the metabolite [9, 10] or may synthesize and secrete its own toxic metabolites [11].

Typical genome of a filamentous fungus harbors dozens of gene clusters putatively involved in the synthesis of secondary metabolites. Many of these clusters appear to be silent *in vitro*; the corresponding metabolic products are mostly unknown [2]. It only recently became apparent that the expression of many of these gene clusters is induced or suppressed by biotic interactions [12–15]. Reoccurring interactions drive the selection for antibiotic production, as recently demonstrated by competition-based laboratory evolution of a *Streptomyces* sp. [16]. The genetic repertoire for secondary metabolites production and its control thus reflects the adaptation of fungus to its ecological niche.

Several studies have addressed the effect of fungal interactions on mycotoxin production. Interaction with other fungi often inhibited but only infrequently stimulated mycotoxin synthesis [15, 17–19]. Estrada et al. [20] compared the growth and metabolic profiles of *Fusarium verticillioides* and *Ustilago maydis* interacting on agar plates. They reported that *Fusarium* suppressed the growth of *Ustillago* and induced production of certain metabolites by *Ustillago*, which were hypothesized to function as antibiotics against *Fusarium*. Surprisingly, *Fusarium* biomass was greater when the fungus was growing in dual culture with *Ustillago* than when growing alone. The authors suggested that *Fusarium* might have acted as a mycoparasite, i.e., it might have killed and then consumed *Ustillago* cells, but the hypothesis has not been supported by data so far.

In the present work, we investigate the induction, inhibition and degradation of secreted fungal metabolites in mixed fungal cultures. A common saprophyte (*Aspergillus niger*, *A.n.*), a plant pathogen (*Fusarium verticillioides*, *F.v.*), and a mycoparasite with a saprophytic life phase (*Clonostachys rosea, C.r.;* syn. *Gliocladium roseum* [21]) were selected as representatives of Ascomyceta with different lifestyles which are known to grow well in minimal medium with organic nitrogen source. The comparison of dual cultures in all combinations was used to determine whether competitive fitness is “congruent” (i.e., whether relative fitness of strains A, B and C following the patterns A > B and B > C implies A > C) or “incongruent” (i.e., whether relative fitness of strains A, B and C may follow the patterns A > B, B > C, and C > A). Furthermore, we determined the extent to which metabolites secreted in single cultures are induced or suppressed in dual cultures; the extent to which new metabolites are synthesized in dual cultures; and how the production of mycotoxin fumonisin by *F.v.* is affected by the mycoparasite *C.r.*

## Results

### Fungal biomass in dual cultures

**Fig. 1:**
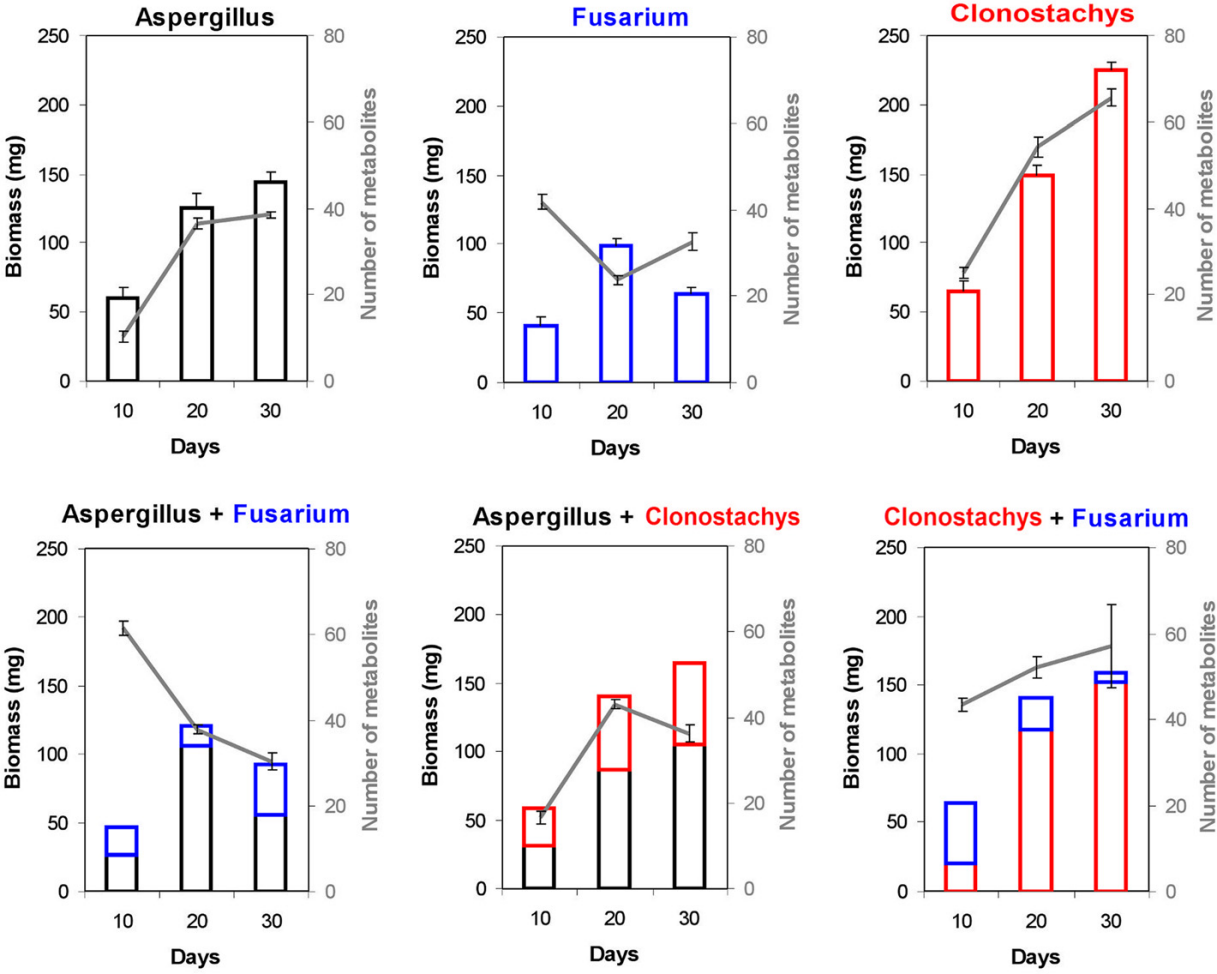
Fungal biomass and metabolic diversity in single and dual cultures *Aspergillus niger, Fusarium verticillioides,* and *Clonostachys rosea* over time. Number of metabolic signals detected by HPLC-MS (grey lines) and the fungal biomass (bars) for single and dual cultures of *Aspergillus niger*, *Fusarium verticillioides,* and *Clonostachys rosea* after 10, 20, and 30 days of incubation are shown. Standard errors are indicated for the biomass in single cultures and for the number of metabolic signals. Standard errors for the biomass of fungi in dual cultures were smaller than 9 mg for all cultures and harvest time except *A.n.* in co-culture with *F.v.* at 20 d (13.6 mg) and *C.r.* in co-culture with *F.v.* at 30 d (10.2 mg)

*A.n.*, *F.v.*, and *C.r.* were grown for 10, 20, and 30 days in single and dual cultures without agitation. Mycelia were harvested, dried, and weighed (Table 1) and pH of the supernatant was determined (Table 2). The contribution of each species to the biomass in dual cultures was determined by DNA analysis (Additional file 1: Table S1). The development of fungal biomass over time is shown in Fig. 1. *C.r.* produced the largest biomass at all times in single cultures, whereas the biomass of *F.v.* declined after 20 d, indicating autolysis (Fig. 1). The effects of competition on the fitness of interacting fungi, estimated by comparing their biomass in single and dual cultures, are shown in Table 3. The biomass of *A.n.* dominated in both dual cultures over its competitor. A strong acidification of the medium by *A.n.* may account for its superior growth (Table 2). In spite of the apparent dominance of *A.n.* over *F.v.*, the growth suppression in *A.n.* in co-cultures with *F.v.* as compared to single cultures of *A.n.* was similar (10 days) or even larger (30 days) than the growth suppression experienced by *F.v.* in co-culture with *A.n.*, as indicated by the indices of competitive fitness (Table 3). Negative value for *A.n.* and positive value for *F.v.* indicate that *F.v.* gained advantage over *A.n.* or suffered less inhibition in dual cultures of *A.n./F.v.* (Methods, section Estimation of the effect of competition on fitness).

**Table 1:** Total fungal biomass in single and dual cultures

Fungus	Fungal biomass ± standard deviation (mg)		
	Day 10	Day 20	Day 30
A.n.	60 ± 9	126 ± 11	144 ± 8
F.v.	41 ± 6	98 ± 6	64 ± 5
C.r.	65 ± 8	149 ± 8	225 ± 6
A.n. + F.v	46 ± 9	121 ± 11	93 ± 10
A.n. + C.r.	58 ± 8	140 ± 10	164 ± 10
F.v. + C.r.	64 ± 8	141 ± 6	159 ± 9

**Table 2:** Acidity of culture supernatants

Fungus	pH [mean ± SD]		
	Day 10	Day 20	Day 30
A.n.	1.77 ± 0.04	1.78 ± 0.02	1.88 ± 0.02
C.r.	5.47 ± 0.04	5.72 ± 0.02	6.13 ± 0.05
F.v.	5.47 ± 0.04	5.07 ± 0.05	6.03 ± 0.05
A.n. + F.v	3.07 ± 0.09	2.25 ± 0.04	2.32 ± 0.02
A.n. + C.r.	1.83 ± 0.09	1.87 ± 0.05	2.12 ± 0.1
F.v. + C.r.	5.47 ± 0.04	5.13 ± 0.05	6.25 ± 0.04

**Table 3:** Fitness of fungal species in single and dual cultures of *Aspergillus niger* (A.n.), *Fusarium verticillioides* (F.v.), and *Clonostachys rosea* (C.r.)

Days	Interacting fungi	Relative biomass in dual culture [rB_i(d,j)_]		Relative biomass in single culture [rB_i(s,j)_]		Growth rates in single cultures	Index of competitive fitness [rFi(j)]		
	i + j	i	j	i	j		A.n.	F.v.	C.r.
10	A.n. + F.v.	1.12	0.88	1.19	0.81	C.r. > A.n. > F.v.	−0.06	0.09	
	A.n. + C.r.	1.07	0.93	0.96	1.04		0.11		−0.11
	F.v. + C.r.	1.35	0.65	0.77	1.23			0.75	−0.47
20	A.n. + F.v.	1.76	0.24	1.12	0.88	C.r. > A.n. > F.v.	0.57	−0.73	
	A.n. + C.r.	1.24	0.76	0.92	1.08		0.35		−0.3
	F.v. + C.r.	0.33	1.67	0.8	1.2			−0.59	0.39
30	A.n. + F.v.	1.2	0.8	1.38	0.62	C.r. > A.n. > F.v.	−0.13	0.29	
	A.n. + C.r.	1.28	0.72	0.78	1.22		0.64		−0.41
	F.v. + C.r.	0.09	1.91	0.44	1.56			−0.8	0.22

### Diversity of secreted metabolites

The number of metabolites detected in single and dual cultures over time is shown in Fig. 1. The total number of metabolites extracted from the supernatants of single cultures of *A.n.* and *C.r.* increased over time in parallel with the increase in biomass. This trend was not apparent in *F.v.* cultures, probably because the mycelium underwent autolysis between day 20 and 30. In dual cultures of *A.n/F.v.*, the number of detectable metabolites decreased by 50 % during the cultivation period, indicating that metabolites secreted at an early stage were taken up and possibly catabolized by aging mycelia. This trend was not apparent for the number of metabolites detected in the supernatants of dual cultures of *A.n./C.r.* and *C.r./F.v*.

**Fig. 2:**
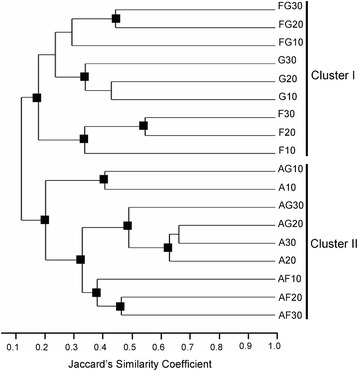
Comparison of metabolic profiles of single and dual cultures of *Aspergillus niger, Fusarium verticillioides,* and *Clonostachys rosea.* Cluster analysis was performed by UPGMA using the Jaccard’s similarity coefficients (coph. coeff. = 0.906) for HPLC-MS profiles of ethanol extracts of culture supernatants. Nodes denoted as ■ were supported by >75 % bootstraps for 2000 replicates. Single cultures: A - *Aspergillus niger* (*A.n.*); F - *Fusarium verticillioides* (*F.v.*); G - *Clonostachys rosea* (*C.r.*). Double cultures: AF - *A.n./F.v.*; AG - *A.n./C.r.*; FG - *F.v./C.r.* Incubation time (10, 20, and 30 days) is shown in the second part the labels of metabolic profiles

Similarities among aligned metabolic profiles for all cultures were investigated by hierarchical clustering. UPGMA based on Jaccard’s similarity indices revealed that the profiles split into two major clusters (Fig. 2). Cluster I consisted of metabolites produced by single cultures of *F.v.* and *C.r.* and their dual cultures at all times. Cluster II consisted of *A.n.* alone and in dual culture with *F.v.* and *C.r.* at all times. The results show that metabolic profiles were less affected by culture age than by interacting species. Cluster II also illustrates the dominance of *A.n.* metabolites in dual cultures with *C.r.* and *F.v.* Subclusters are also evident in Fig. 2. After 20 and 30 days, the metabolic profiles of *F.v./C.r.* dual cultures clustered separately from those of single cultures of *F.v.* or *C.r.*, indicating that the metabolic profiles of the dual cultures were dominated by newly formed “interaction-specific” metabolites (defined further in the next section). A similar pattern was evident in the interaction between *A.n.* and *F.v.* in that the metabolic profiles of *F.v./A.n.* dual cultures formed a subcluster distinct from those of *A.n.* and *F.v.* single cultures at all times. This was, however, not true for the interaction between *A.n.* and *C.r.*, which was dominated by *A.n.* Taken together, these results indicate strong effects of fungal interactions on the composition and complexity of secreted metabolites. These effects are further dissected in the following sections.

### Suppression and stimulation of metabolite production by fungal interactions, transformation of secreted metabolites by competing fungi

We normalized signal intensities by dividing peak areas with the biomass of the fungus that produced the signals, and we then compared the normalized signal intensities between single cultures and dual cultures to identify metabolites suppressed, stimulated, and newly formed in dual cultures. Metabolites with normalized intensities at least 10-times lower in dual cultures as compared to single cultures were designated suppressed. Some of the metabolic signals observed in single cultures were not found in dual cultures, and we refer to this phenomenon as complete suppression. The number of metabolic signals suppressed in dual culture is shown in Table 4. It is evident that among the three interacting species, *A.n.* had the greatest ability to suppress the metabolites produced by the other fungi in dual cultures. Apart from direct suppression of metabolite synthesis, recognition of *A.n.* as a competitor followed by re-programming of metabolism may account for the suppression of metabolite production by the other fungi. The metabolites of *A.n.* were the least suppressed by the other fungi. As shown in Table 4, some metabolites were suppressed specifically in dual cultures with one competitor while others were suppressed by both competitors.

**Table 4:** Suppression of secreted metabolites in dual cultures of *Aspergillus niger* (*A.n.*), *Fusarium verticillioides* (*F.v.*), and *Clonostachys* rosea (*C.r.*). Suppressed metabolites were defined as metabolites with normalized signal intensities in dual cultures at least 10-times lower as compared to single cultures. Metabolites transformed or catabolized by interacting species were not included

Producer	Competitor	Number of suppressed metabolites^a^					
		Day 10		Day 20		Day 30	
		-ve^b^	+ve^b^	-ve^b^	+ve^b^	-ve^b^	+ve^b^
A.n.	F.v.	6	7	7	15	9	28
	C.r.	2	0	2	1	5	11
	F.v. & C.r.^c^	2	0	1	1	2	8
F.v.	A.n.	31	56	17	47	13	39
	C.r.	20	34	13	20	13	35
	A.n. & C.r.^c^	18	21	8	13	8	19
C.r.	A.n.	28	31	32	68	36	103
	F.v.	23	19	25	42	24	36
	A.n. & F.v.^c^	21	16	22	37	23	26

^a^Criteria: minimum intensity for negative ionization 75,000 cpm, for positive ionization 500,000 cpm; MCQ 0.9; relative standard deviation among replicates < 1; change fold-factor < 0.1

^b^Negative and positive ionization modes are indicated by -ve and +ve, respectively

^c^Number of metabolites suppressed by both competitors

Some metabolites accumulated at higher concentrations in the supernatants of dual cultures than in those of single cultures. When signal intensity normalized by biomass increased 10-times or more in a dual culture relative to single culture, the metabolite was referred to as stimulated. Table 5 shows the number of stimulated metabolites for all producer/competitor combinations. Generally, the number of stimulated metabolites was low; no stimulated metabolites were detected in dual cultures of *A.n.* and *C.r.* at any sampling time.

**Table 5:** Secreted metabolites stimulated in dual cultures of *Aspergillus niger* (*A.n.*), *Fusarium verticillioides* (*F.v*.)*,* and *Clonostachys rosea* (*C.r.*). Induced metabolites were defined as metabolites with normalized signal intensities at least 10-times larger in dual cultures as compared to single cultures

Producer	Competitor	Number of induced metabolites^a^					
		Day 10		Day 20		Day 30	
		-ve^b^	+ve ^b^	-ve^b^	+ve ^b^	-ve^b^	+ve ^b^
A.n.	F.v.	1	0	0	0	0	2
F.v.	A.n.	0	4	0	2	0	0
F.v.	C.r.	0	1	0	5	1	8
C.r.	F.v.	0	1	0	2	0	0
A.n.	C.r.	0	0	0	0	0	0
C.r.	A.n.	0	0	0	0	0	0

^a^Criteria: minimum intensity for negative ionization 75,000 cpm, for positive ionization 500,000 cpm; MCQ 0.9; relative standard deviation < 1; change fold-factor > 10

^b^Negative and positive ionization modes are indicated by -ve and +ve, respectively

Many metabolites detected in the supernatants of dual cultures were not found in any single culture. The origin of these metabolites is unknown; we refer to them as new, interaction-specific metabolites (Table 6). The largest number of new metabolites was detected in *A.n.*/*F.v.* dual cultures followed by *F.v.*/*C.r.* dual cultures sampled on day 10. Surprisingly, many new metabolites detected on day 10 were unstable or were degraded or transformed by fungal cultures, because fewer interaction-specific metabolites were detected on day 20 and day 30 than on day 10.

**Table 6:** Interaction-specific metabolic signals obtained from dual cultures of *Aspergillus niger* (*A.n.*), *Fusarium verticillioides* (*F.v.*), and *Clonostachys rosea* (*C.r.*). New metabolites were defined as signals found in dual cultures but not in single cultures

Interacting fungi	Number of interaction-specific metabolites^a^					
	Day 10		Day 20		Day 30	
	-ve^b^	+ve^b^	-ve^b^	+ve^b^	-ve^b^	+ve^b^
A.n. + F.v	37	68	1	10	4	7
A.n. + C.r.	7	23	6	17	4	18
F.v. + C.r.	19	39	17	37	7	31

^a^Criteria: minimum intensity for negative ionization 75,000 cpm; for positive ionization 500,000 cpm; MCQ 0.9. Products of biotransformation were excluded

^b^Negative and positive ionization modes are indicated by -ve and +ve, respectively

To determine whether transformation of metabolites produced by a competitor was responsible for the accumulation of new metabolites in dual cultures, a mycelium exchange experiment was performed. Metabolites extracted from the supernatant of each fungus grown in single culture for 10 and 20 days were incubated with washed mycelium (10 days old) of the other fungi. After 3 days, metabolic profiles of the supernatants were recorded to identify metabolites that were absorbed, catabolized, and/or transformed by the mycelium. The results (Table 7) showed that accumulation of new metabolites in dual cultures was not or only to a minor extent caused by biotransformation. Specifically, no metabolite produced by *A.n.* was transformed by the other two fungi. Most new metabolites found in dual cultures therefore appear to originate from biosynthetic pathways that were silent in single cultures.

**Table 7:** Secreted fungal metabolites transformed by another fungus. Ethyl acetate extract of culture supernatant of fungus labeled as metabolite producer was incubated with washed mycelium of another fungus and the number of metabolic signals that disappeared was counted

Metabolite producer	Fungus responsible for biotransformation	Number of metabolites transformed ^a^			
		Metabolites from day 10		Metabolites from day 20	
		- ve^b^	+ ve^b^	- ve^b^	+ ve^b^
A.n.	F.v.	0	0	0	0
	C.r.	0	0	0	0
F.v.	A.n.	2	2	4	5
	C.r.	0	0	0	0
C.r.	A.n.	1	0	3	5
	F.v.	0	0	0	0

^a^Criteria: minimum intensity for negative and positive ionization 75,000; MCQ 0.9; relative standard deviation < 1; changefold factor < 0.1

^b^Negative and positive ionization modes are indicated by -ve and +ve, respectively

**Fig. 3:**
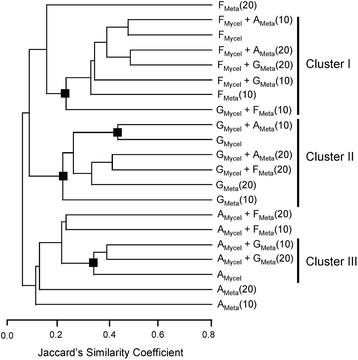
Biotransformation of secreted fungal metabolites by mycelium of a different fungus. Ethyl acetate extracts of supernatants of 10 and 20 days old culture of one species were incubated with mycelium of a second species to determine whether the metabolites of one fungus can be transformed by another fungus. The resulting metabolic profiles were subjected to cluster analysis by UPGMA using Jaccard’s similarity coefficients (coph. coeff = 0.944). Nodes denoted as ■ were supported by >75 % bootstraps for 2000 replicates. A_Mycel_, F_Mycel_, G_Mycel_: metabolic profiles of supernatants of washed mycelium of *Aspergillus niger* (*A.n.*), *Fusarium verticillioides* (*F.v.*), *Clonostachys rosea* (*C.r.*), respectively; A_Meta_, F_Meta_, G_Meta_: metabolic profiles of supernatants of single cultures *A.n.*, *F.v.*, and *C.r.*, respectively; X_Mycel_ + Y_Meta_: metabolic profile of the supernatant after incubation of mycelium X with metabolites extracted from supernatant of Y

Cluster analysis of aligned metabolic profiles for all cultures revealed that, regardless of the metabolites that were added to the culture medium, metabolic profiles of media after incubation were dominated by the metabolites produced by the species that was growing in the medium (Fig. 3). Feeding metabolites produced by one fungus to another fungus was not sufficient to generate a large number of new metabolites observed in dual cultures (Table 6) that caused the sub-clustering of metabolic profiles shown in Fig. 2. In line with the results of the mycelium exchange experiment (Table 7), activation of biosynthetic pathways that were inactive in single cultures rather than biotransformation of metabolites accumulated in the medium was responsible for the occurrence of new metabolites in dual cultures.

### Fumonisins in dual cultures of *F. verticillioides* with *C. rosea*

**Fig. 4:**
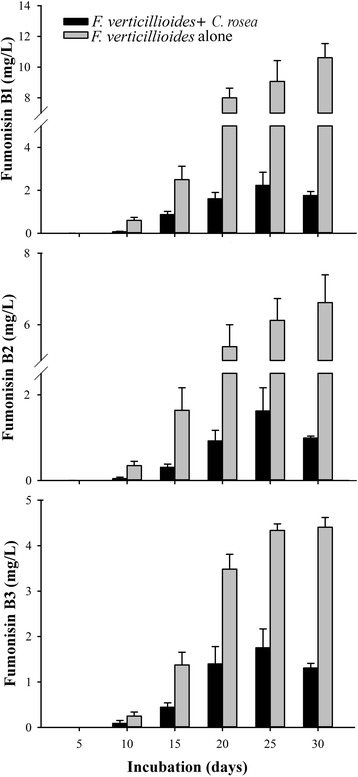
Accumulation of fumonisins in pure cultures of *F. verticilloides* and dual cultures of *F. verticillioides* with *C. rosea*. The fungi were incubated in GM7 medium grown in the dark at 21 °C. Error bars show the standard error of mean. Concentrations of fumonisins in the supernatants in the course of time were determined by HPLC-MS/MS

*F.v.* is known to produce carcinogenic mycotoxins fumonisin B1 (FB1), fumonisin B2 (FB2) and fumonisin B3 (FB3). All three fumonisins were found in all cultures of *F.v*., as proven by comparing retention time and MS^2^ spectra of authentic standards with that of signals found for m/z of 722 (FB1) and 706 (FB2 and FB3) in positive ionization mode. FB1 was the most abundant fumonisin in all cultures. As compared to pure cultures of *F.v*., co-incubation with *C.r.* reduced fumonisin production at all times (Fig. 4). After 30 days, the amount of FB1 was 5-times lower in *F.v.*/*C.r.* dual cultures as compared to single cultures of *F.v.*; the amount of FB2 was 7-times lower, and the amount of FB3 was 4-times lower. We speculated that the reason for the reduction of fumonisin concentration in dual cultures of *F.v.* with *C.r.* was degradation of fumonisins by *C.r.* We therefore incubated pure FB1 with *C.r.* No degradation of FB1 by *C.r.* was found. The biomass of *C.r.* was not affected by FB1 up to a concentration of 160 μg/ml in the medium (data not shown).

## Discussion

### Competitive fitness

Densitometry of species-specific DNA fragments after agarose electrophoresis, which is an established method for the quantification of DNA standards [22], was used to partition the dry weight of total fungal mycelium from dual cultures into contributions of each species. The low standard deviations for replicated cultures demonstrated an excellent reproducibility of the method, which would not be achievable by real-time PCR. The biomass of both *A.n.* and *C.r.* in single cultures increased over the entire cultivation period (Fig. 1). The growth of *F.v.* was slower and the culture apparently underwent autolysis after day 20, perhaps because the phytopathogen and endophyte is not adapted to saprophytic growth under the conditions used. The biomass of *C.r.* in single culture was higher than that of *A.n.* and *F.v.* at all times, indicating a high saprophytic fitness of this species even though *C.r.* is known as a mycoparasite [23].

The DNA of *F.v.* in dual cultures with *C.r.* was strongly reduced at day 20 and nearly disappeared at day 30, suggesting that the mycelium of *F.v.* was destroyed and/or consumed by *C.r*. Interestingly, *F.v.* generated similar amounts of biomass in dual cultures with *C.r.* as in single cultures (cp. Table 2 and Additional file 1: Table S1) at day 10, while the growth of *C.r.* was strongly suppressed at this time point (Fig. 1). As the indices of competitive fitness for dual cultures *C.r./F.v.* at day 10 show (Table 3), *F.v.* built more biomass than in single cultures while *C.r.* built less biomass than in single cultures. This resembles the result of Estrada et al. [20] with *F.v.* and *Ustilago maydis* The unexpected fitness gain of *F.v.* in dual cultures with *U. maydis* and *C.r.* may be explained by access to nutrients or growth factors released by co-cultivated fungi; the phenomenon deserves further investigation. The temporary growth retardation of *C.r.* may have resulted from antibiosis exerted by *F.v.* or from physiological control of growth rate; slowing down metabolic rate and replication was shown to induce an “antibiotic-tolerant” state in *Mycobacterium tuberculosis* [24]. We speculate that during the first 10 days, *C.r.* activated mechanisms enabling the fungus to cope with fungitoxic metabolites produced by *F.v.* and potentially involved in interference competition; these mechanisms may have included the reduction of growth rate*.* A high number of mutually suppressed metabolites (Table 4) as well as interaction-specific metabolites (Table 6) in dual cultures of *C.r./F.v.* corroborate important role of secreted metabolites in this interaction. At later growth stages, the biomass of *F.v.* declined while *C.r.* continued growing. Part of the decline of *F.v.* biomass can be explained by autolysis. However, slower biomass decline was observed in single cultures of *F.v.* and no decline occurred in co-culture with *A.n.* We therefore suggest that active mycoparasitism of *F.v.* by *C.r.* might have been involved in the decline of biomass of F.v. in *C.r./F.v.* cultures*.*

*C.r.* accumulated less biomass in dual culture with *A.n.* than with *F.v.* (Fig. 1). The growth of both fungi was reduced relative to single cultures but the suppression was much more pronounced for *C.r.* The mycoparasite generated more biomass than *A.n.* in single cultures but less biomass than *A.n.* in dual cultures; its index of competitive fitness in co-cultures with *A.n.* remained below -0.3 at all times. Acidification of the growth medium by *A.n.* (Table 2) might account for the suppression of *C.r.* in co-culture with *A.n.* because pH of supernatants of *A.n./C.r.* cultures was similar to the supernatants of single cultures of *A.n.*

The co-incubation with *A.n.* apparently delayed the senescence and autolysis of *F.v.* mycelium as compared to single cultures (Fig. 1). Given that *A.n.* outcompeted *C.r.* and that *C.r.* essentially destroyed *F.v.*, it was surprising that *A.n./F.v* dual cultures did not unequivocally show a dominance of *A.n.* over *F.v.* The biomass of *A.n.* was larger than that of *F.v.* at all times (Fig. 1) but the growth suppression was similar for both species at day 10 and was larger for *A.n.* than for *F.v.* at day 30, resulting in a higher index of competitive fitness for the latter species. In contrast to *C.r.*, *F.v.* was apparently able to counteract acidification of the medium caused by *A.n.* (Table 2); the comparison of fungal biomass and pH of the supernatants of dual cultures indicates that acidification of growth medium is a mechanism of interference competition exerted by *A.n.* If some nutrients became less accessible to *C.r.* at a low pH while remaining accessible to *A.n.* which is adapted to acidic conditions, exploitative competition may have occurred. Induction of costly defense responses might have contributed to the suppression of biomass accumulation in co-cultures, too. It was not possible in our experiments to distinguish between growth suppression caused by interference competition and growth reduction due to the costs of inefficient defense. Monitoring metabolic costs of all pathways and the separation of growth suppression effects of metabolites and enzymes involved in interference competition will be needed to fully explain growth suppression of fungi in dual cultures.

At days 10 and 20, the rankings for competitive fitness were congruent (if A > B and B > C, then A > C). The rankings were *A.n.* ≈ *F.v.* > *C.r.* at day 10 and *A.n.* > *C.r. > F.v.* at day 20. At day 30, however, the rankings were incongruent in that *F.v.* > *A.n.*, *A.n.* > *C.r.*, and *C.r.* > *F.v.* This situation is reminiscent of non-transitive systems of three competing species with a rock–paper–scissors relationship [25]. Our finding indicates that fungal competitive fitness depends on the competitor, which is in line with observations of Losada et al. [11] for competing *Aspergillus* species grown on agar plates. Differences in the ranking among time points indicate that physiological adaptation altered the competitive fitness of interacting fungal strains in the course of incubation. Understanding how it happened will require monitoring of fungal biomass at short time intervals.

### Metabolic diversity

According to recent research, the synthesis of novel metabolites can be activated by the co-culturing of fungi with other microbes [12–14]. These research efforts focused on the recovery of new structures with potentially useful properties, and the effect of biotic interactions on metabolic diversity was not systematically studied. Metabolic fingerprints generated by HPLC-MS in our work revealed a dramatic increase in the diversity of secreted metabolites in dual fungal cultures. Some of these signals may have resulted from biotransformation that escaped detection in mycelium exchange experiments; many of them, however, are likely to have originated from new structures and/or from derivatives of known metabolites that have not yet been isolated from these organisms. The low ratio of the number of known secondary metabolites to the number of relevant metabolic pathways found in sequenced fungal genomes is consistent with this hypothesis [2].

Intensity of competition has been shown to modulate plant metabolic diversity [26], although the evidence for direct involvement of plant metabolites in plant–plant interactions is limited. Simulation of microbial interactions in spatially structured communities suggested that antibiosis maintains microbial diversity on an evolutionary time scale [27]. Model predictions were confirmed by *E. coli* populations as long as the interactions and strain dispersal were limited in space [25]. Our results suggest an extension of the paradigm of competition-driven maintenance of species/strain diversity to include the competition-driven maintenance of the diversity of secondary metabolites that a fungal species is able to produce. Based on the large increase in the number of metabolic signals obtained from interacting cultures (Table 6), we hypothesize that re-occurring competition has selected for fungal lineages that respond to biotic challenges by synthesizing a large set of metabolites with different modes of action, increasing the chance that some of these metabolites will suppress the competitor. Research addressing the diversity and composition of secreted metabolites during interspecific interactions among fungi in ecological context is needed to reinforce or reject these hypotheses and advance our understanding of fungal ecological chemistry beyond specific effects of individual metabolites.

### Stimulation, suppression, and degradation of secondary metabolites in dual cultures

Contrary to our expectation, only a few of secreted metabolites found in single cultures were significantly stimulated (i.e., produced in ≥ 10-times amounts) in dual cultures (Table 5). In line with the observation of a large number of new metabolites in dual cultures (Table 6), this finding suggests that the synthesis of metabolites involved in interactions with other species is fully suppressed in the absence of these interactions. A tight control of secondary metabolite synthesis is likely to result from the selection for the minimization of metabolic costs. Some interaction-specific metabolic signals may also have originated from intracellular metabolites released after cell lysis.

Many secreted metabolites accumulated in lower amounts in dual cultures than in single cultures (Table 4). This suppression could be caused by the inhibition of synthesis or secretion, by enzymatic transformation or degradation in the medium, or by uptake of the metabolite by the interacting fungus or adsorption on its cell wall. It is evident from Table 4 that the number of secreted secondary metabolites suppressed in dual cultures was affected by the producers as well as the competitors. A particular metabolite from fungus A may be suppressed by fungus B only, by fungus C only, or by both fungus B and C. Among the three fungal species studied, *A.n.* metabolites were the least suppressed. This observation was consistent with biomass data, which indicated that in dual cultures *A.n.* outcompeted the other two species.

Most metabolic signals observed on day 10 and day 20 had disappeared by the next time point. We hypothesize that catabolism by the producers of the metabolites themselves was the cause. Fungi might be able to take-up their own secondary metabolites and use them as a source of energy. The phenomenon has rarely been documented [28, 29]. Uptake and catabolism of own secreted metabolites might be a widespread capability of filamentous fungi. The ability to re-use metabolic resources might increase fungal fitness particularly under starvation conditions.

### Fumonisin accumulation in dual cultures of *Fusarium verticillioides* with *Clonostachys rosea*

Fumonisins contaminating maize grain endanger the health of consumers and farm animals. Strong decline of fumonisin content in dual cultures of *F.v.*/*C.r.* as compared to pure cultures of *F.v.* raised a question whether the mycoparasite is able to degrade fumonisins enzymatically, as it is known to detoxify other mycotoxins [10], which would offer an opportunity to exploit the activity for decontamination of plant products [30]. When pure fumonisin B1 was added into growth medium, however, no degradation was observed.

When *C.r.* did not degrade fumonisins, why was fumonisin accumulation reduced in dual cultures *F.v./C.r.* as compare to pure cultures of *F.v.*? After day 10, *F.v.* biomass declined, apparently due to destruction of the mycelium by *C.r.* (Fig. 1). We have not normalized fumonisin production by the biomass of *F.v*. because degradation of *F.v.* would lead to an overestimation of fumonisin productivity. However, no destruction of *F.v.* biomass occurred during the first 10 days. In spite of that, significantly less fumonisins accumulated in dual cultures *F.v.*/*C.r.* at day 10 as compared to pure cultures of *F.v.* (Fig. 4). As discussed above in section “Competitive fitness”, *F.v.* actually gained more biomass in co-cultures with *C.r.* as compared to pure cultures. Apparently co-cultivation with *C.r.* suppressed fumonisins production in *F.v.*, which can be regarded as an advantageous side-effect of the application of *C.r.* in the biological control of *F.v*. The effect of co-incubation of *F.v.* with other fungi on fumonisin production has been mainly studied in the laboratory of V. Sanchis in Lleida [18, 19, 31, 32]. Depending on the competing species and incubation conditions, fumonisin production was stimulated or suppressed but the effects were less dramatic than in our dual cultures with *C.r.* Fumonisin production was suppressed by co-incubation with *Trichoderma* sp. [18, 33] and sometimes by other *Fusarium* spp. [18] but not by *Aspergillus parasiticus* [19]. Estrada et al. [20] observed slight suppression of fumonisin levels normalized by biomass in interaction between *F.v.* and *U. maydis*. They speculated that fumonisins may have limited the growth of *U. maydis* in dual cultures with *F.v.* Except for a single publication from the South African Medical Research Council [34], the effect of fumonisin on fungi has not been studied systematically. Fumonisin B1 has not affected the growth of *C.r.* in our experiments, indicating that fumonisins are not involved in the defense of *F.v.* against mycoparasites. This result corroborates the conclusion of Marin et al. [31] that fumonisins are not involved in fungal antagonism. Biological function of fumonisins thus remains puzzling.

*A. niger* was reported to produce fumonisins B2 and B4. We have not found fumonisins in single and dual cultures of *A. niger* that did not contain *F. verticillioides*, which can probably be accounted for by high water activity in our cultures. Low water activity is required for fumonisin production by *A. niger* [35, 36].

## Conclusions

Competitive fitness in pairwise interactions among fungi varied in the course of interaction indicating adaptation. Furthermore, competitive fitness was incongruent, showing that it was controlled by species-specific factors. In aging cultures all three species were able to catabolize most of their secreted metabolites. Interaction among fungi increases the diversity of secreted metabolites accumulating in the growth medium, corroborating the hypothesis that secondary metabolite production plays a key role in interference competition. *C. rosea* suppressed the synthesis of fumonisins by *F. verticillioides* but fumonisins did not affect the growth of *C. rosea* nor were they degraded by the mycoparasite. Understanding how interacting fungi recognize each other and how this recognition controls secondary metabolite synthesis is a major task for research in fungal chemical ecology.

## Methods

### Fungi and media

The fungi studied included the saprophyte *Aspergillus niger* AvT 14.203, the mycoparasite *Gliocladium roseum* DSM 62726 (syn. *Clonostachys rosea*; obtained from Deutsche Sammlung von Mikroorganismen und Zellkulturen, Braunschweig, Germany), and the phytopathogen *Fusarium verticillioides* FRC M-8114 (obtained from the Fusarium Research Center, PA, USA). The fungi were maintained on potato dextrose agar plates. Potato dextrose agar was purchased from Carl Roth GmbH (Karlsruhe, Germany) and prepared according to the manufacturer’s instruction. For the experiments, the fungi were grown on agar plates containing GM7 medium which contains asparagin as nitrogen source [10], and spore suspensions were made in sterile-distilled water. For liquid cultures, GM7 medium was prepared in the same way except that glucose was autoclaved separately and no agar for solidification was used.

### Chemicals

All chemicals used were of “pro analysis” quality. Methanol (gradient quality; Fisher Scientific, Schwerte, Germany), acetonitrile (gradient quality; VWR, Darmstadt, Germany), and acetic acid (LCMS grade; Fluka/Sigma-Aldrich, St. Louis, USA) were used for the mobile phases in HPLC. TE buffer contained 10 mM Tris and 1 mM EDTA; the pH was adjusted to 8.0. Analytical standards of fumonisins B1, B2 and B3 were purchased from Biopure (Tulln, Austria).

### Culture conditions

Four replicates of single and dual cultures of *Aspergillus niger* (*A.n.*), *Fusarium verticillioides* (*F.v.*), and *Clonostachys rosea* (*C.r.*) were grown in the dark at 21 °C in 100-ml Erlenmeyer flasks containing 30 ml of GM7 medium inoculated with 100 μL of a spore suspension that contained 10^5^ spores/ml. For dual cultures (designated *F.v.*/*C.r.*, *F.v.*/*A.n*., and *A.n.*/*C.r.*), an equal number of spores from two species was inoculated. Cultures were grown without agitation to prevent the development of mycelial lumps, and the medium in the flask formed only a thin layer (13 mm), which insured its saturation with oxygen. Fungal mycelium from single and dual cultures was harvested by filtration after 10, 20, and 30 days. The harvested mycelium was freeze-dried. For the fumonisin biotransformation experiment, *C.r.* cultures in GM7 amended with fumonisin B1 at 5 mg/L was incubated at 21 °C. Four cultures were harvested after 5, 10, 15, 20, 25 and 30 days and the supernatants were analyzed for fumonisin content.

### Determination of fungal biomass in dual cultures by densitometry of species-specific fragments of 28S genes

#### Principle

Total biomass of both fungi in a dual culture was determined as the weight of the freeze-dried mycelium. Because real-time PCR possesses too large an error margin to reveal small differences in the biomass of interacting fungi, we used densitometry of species-specific restriction fragments of co-amplified 28S RNA genes to calculate the proportion of each species to the total biomass. For this purpose, a 900-bp fragment of the 28S RNA gene was amplified using primers common to all three fungal species in the study and was digested by restriction enzymes to produce species-specific fragments. Relative quantities of the fragments, corrected for fragment length, were used to calculate the absolute biomass of each species. The lengths of the restriction fragments of the amplified portion of the 28S RNA gene for *A.n.*, *F.v.*, and *C.r.* used in this study are listed in Table 8; fragments differing in the size by at least 53 bp were used for quantification. Fungal biomass in single cultures was determined as the dry weight of the mycelium.

**Table 8:** Restriction enzymes and DNA fragments used for species-specific biomass determination

Species combination (restriction enzymes)	Fragment size (bp)		
	*Aspergillus niger* (A.n.)	*Fusarium verticillioides* (F.v.)	*Clonostachys rosea* (C.r.)
A.n./F.v. (MseI)	617^a^ + 307	550^a^ + 300 + 57	-
A.n./C.r. (MseI + ApoI)	500^a^ + 193 + 117 + 87 + 27	-	603^a^ + 271 + 27
F.v./C.r. (MseI)	-	550^a^ + 300 + 57	603^a^ + 298

^a^Fragments used for quantification

#### Genomic DNA isolation from single and dual cultures

Lyophilized mycelium was ground in a ball mill (Mixer Mill MM 200, Retsch, Hann, Germany) in a 2-ml tube with five wolfram carbide spheres (diameter 3 mm) for 30 s at maximum speed. For DNA extraction, a variant of the CTAB method was used as described earlier [22]. The quality and quantity of DNA was assessed by electrophoresis in 0.8 % (w/v) agarose gels (Cambrex, Rockland, ME, USA) prepared in TAE buffer (40 mM Tris, 1 mM EDTA, pH adjusted to 8.5 with acetic acid). The electrophoresis was carried out at 4 V/cm for 60 min. The gels were stained with ethidium baromide (2 mg/L) and documented after irradiation with UV light at 360 nm with a digital imaging system (Vilber Lourmat, Marne la Vallee, France). The densitometry was performed using Multi Analyst-Software (BioRad, Hercules, CA, USA). The concentration of fungal DNA was calculated by comparing a dilution series with defined amounts of DNA of lambda phage (methylated, from *Escherichia coli* host strain W3110).

#### PCR amplification and restriction digestion

PCR amplification was carried out in a 25-μL reaction mixture containing 1X PCR buffer (from 10X reaction buffer: 670 mM Tris–HCl, 160 mM (NH_4_)_2_SO_4_, 0.1 % (v/v) Tween-20, pH 8.8 at 25 °C; Bioline, Luckenwalde, Germany), 3 mM MgCl_2_, 0.2 mM of each deoxyribonucleotide triphosphate (Bioline, Luckenwalde, Germany), 0.5 μM of each primer (forward: AACGG CGAGT GAAGC GGCAA and reverse: CTAAT CATTC GCTTT ACCTC ATAAA ACTGA), 0.4 units of Taq DNA polymerase (BIOTaq, Bioline, Luckenwalde, Germany), and 2 μL of template DNA.

The TPersonal thermocycler (Biometra, Göttingen, Germany) was used for PCR amplification. The condition were: an initial denaturation for 2 min at 94 °C; followed by 35 cycles of 30 s denaturation at 94 °C, 30 s annealing at 61 °C, and 60 s elongation at 72 °C; and a final extension for 5 min at 72 °C. The amplification was checked by agarose electrophoresis as described above. PCR products were precipitated with ethanol and dissolved in 25 μL of sterile TE buffer.

A 10-μL volume of purified PCR products was either digested with 5 u of *MseI* (for DNA of *F.v.*/*C.r.* and *F.v.*/*A.n*.) or was doubly digested with 2.5 u each of *MseI* and *ApoI* (for DNA of *A.n.*/*C.r.*) in a 25-μL reaction mixture following the procedure described by the manufacturer of the enzymes (Fermentas, Germany). To obtain DNA bands that could be quantified by densitometry, different dilutions of the digestion products were loaded on agarose gels. For *A.n.*/*C.r.*, 1.7 % (w/v) agarose gel was used, and the separation was carried out at 4 V/cm for 60 min. For *F.v.*/*C.r.* and *F.v.*/*A.n.*, 3 % (w/v) low-MW agarose (Biozyme Scientific, Oldendorf, Germany) was used, and the separation was carried out at 4 V/cm for 210 min. Gels were stained with ethidium bromide (2 mg/L) and visualized and photographed in UV light using a 128-bit camera (Vilber Lourmat, Eberhardzell, Germany).

### Densitometric analysis and biomass estimation

Intensities of DNA bands were determined using Quantity One software version 4.5 (BioRad, Hercules, USA). Relative intensities were normalized by the size of DNA fragments (Table 8) and multiplied by the dry weight of mycelium obtained from dual cultures to determine the biomass of each species.

### Estimation of the effect of competition on fitness

To compare the effect of interaction between three fungi on their biomass in all dual combinations, we calculated the relative biomass of strain i to strain j in single cultures and dual cultures as follows:

1 $$ {\mathrm{rB}}_{\mathrm{i}\left(\mathrm{s},\mathrm{j}\right)}=2*{\mathrm{B}}_{\mathrm{i}\left(\mathrm{s}\right)}/\left[{\mathrm{B}}_{\mathrm{i}\left(\mathrm{s}\right)} + {\mathrm{B}}_{\mathrm{j}\left(\mathrm{s}\right)}\right] $$

2 $$ {\mathrm{rB}}_{\mathrm{i}\left(\mathrm{d},\mathrm{j}\right)}=2*{\mathrm{B}}_{\mathrm{i}\left(\mathrm{d},\mathrm{j}\right)}/\left[{\mathrm{B}}_{\mathrm{i}\left(\mathrm{d},\mathrm{j}\right)}+{\mathrm{B}}_{\mathrm{j}\left(\mathrm{d},\mathrm{i}\right)}\right] $$

where:

- o rB_i(s,j)_ is the relative biomass of strain i compared to strain j in single culture,
- o B_i(s)_ is the biomass of strain i in single culture,
- o B_j(s)_ is the biomass of strain j in single culture,
- o rB_i(d,j)_ is the relative biomass of strain i in dual culture with strain j,
- o B_i(d,j)_ is the biomass of strain i in dual culture with strain j, and
- o B_j(d,i)_ is the biomass of strain j in dual culture with strain i.

The effect of the interaction with strain j on biomass of strain i was determined as the ratio of relative biomass in dual and single cultures:

3 $$ {\mathrm{rF}}_{\mathrm{i}\left(\mathrm{j}\right)}=\left[{\mathrm{rB}}_{\mathrm{i}\left(\mathrm{d},\mathrm{j}\right)}/{\mathrm{rB}}_{\mathrm{i}\left(\mathrm{s},\mathrm{j}\right)}\right]\hbox{-} 1 $$

rF_i(j)_ was designated the index of competitive fitness. In the absence of competition, the biomass of both fungi in a dual culture is expected to grow at the same rate as in single cultures:

4 $$ {\mathrm{rF}}_{\mathrm{i}\left(\mathrm{j}\right)}={\mathrm{rF}}_{\mathrm{j}\left(\mathrm{i}\right)}=0 $$

If and interaction affects growth, rF_i(j)_ and rF_j(i)_ will diverge from 0 in opposite directions:

5 $$ \left(\left({\mathrm{rF}}_{\mathrm{i}\left(\mathrm{j}\right)}<0\right)\mathrm{AND}\left({\mathrm{rF}}_{\mathrm{j}\left(\mathrm{i}\right)}>0\right)\right)\mathrm{OR}\left(\left({\mathrm{rF}}_{\mathrm{i}\left(\mathrm{j}\right)}>0\right)\mathrm{AND}\left({\mathrm{rF}}_{\mathrm{j}\left(\mathrm{i}\right)}<0\right)\right) $$

A positive value of rF_i(j)_ indicates that strain i gains advantage over strain j or suffers less inhibition than strain j in dual cultures of i and j.

### Metabolic profiling of secreted fungal metabolites by HPLC-ESI-MS

#### Extraction of secreted metabolites

Single and dual cultures harvested at three time points were filtered and supernatants were extracted twice with an equal volume of n-hexane for defatting. A 25 ml aliquot of each defatted supernatant was extracted three times with an equal volume of ethyl acetate. Combined extracts were evaporated to dryness under vacuum at 30 °C. The residue was dissolved in 1 ml of methanol/water (1:1), and the solution was filtered through a 0.2-μm Teflon filter (WICOM, Heppenheim, Germany). The solution was immediately subjected to HPLC-MS analysis or was stored at -20 °C.

#### Metabolic profiling by HPLC-MS and fumonisin analysis

For non-targeted metabolite analysis, a reverse-phase HPLC system coupled to an electrospray and ion trap detector 500-MS (Varian) was used as described before [37]. The mobile phase consisted of a binary gradient of 7 mM acetic acid in 95 % water/5 % acetonitrile (A) and 7 mM acetic acid in methanol (B): 0-5 min 90 % A; 5-30 min from 90 % A to 2 % A; 30-38 min 2 % A; and 38-40 min 2 % A to 90 % A at a flow rate of 0.2 ml/min. This was followed by washing and re-equilibration steps. A quality control sample was injected at the beginning, in the middle, and at the end of each sequence to monitor the stability of the method.

Ionization was done by electrospray both in positive and negative mode with the following parameters (negative/positive): needle voltage -4,500 V/+5,000 V, shield voltage -600 V/+600 V, capillary voltage -/+40 V, drying gas (nitrogen) 25 psi (172.5 kPa) at 250 °C, and nebulizing gas (air) 50 psi (345 kPa). The MS analyzer was operated in full-scan mode, mass range *m/z* 100–1000, scan speed 5000 Da/s, and three scans averaged. For data acquisition, MS workstation/MS Data Review 6.9 (Varian) was used. Raw data were converted to netCDF format. Because mass spectrometry signals after ESI originate from molecular ions, the terms MS signals and metabolites are used synonymously in the following text. Fumonisin content was analyzed in culture supernatants without ethyl acetate extraction as described by Adejumo et.al. [38]. Fumonisin concentrations were not normalized by biomass.

#### Data processing

Raw HPLC-MS data were processed with the Component Detection Algorithm (CODA, [39]) implemented in ACD/MS Manager v. 8.0 (Advanced Chemistry Development, Toronto, Canada). The CODA algorithm evaluates the quality of chromatographic peaks by calculating a mass quality index (MCQ) that reflects the similarity between the original mass chromatograms and their smoothed and mean-subtracted versions. Data processing by CODA included smoothing, baseline correction, and peak picking. Peak tables contained the monoisotopic mass (mass to charge ratio for [M-H]^−^ and [M + H]^+^), retention time (Rt), peak area, and MCQ value for each signal that passed the MCQ thresholds of 0.8 and the S/N threshold of 10. Adducts common in our system (sodium in positive ionization and acetyl in negative ionization) were removed manually by checking raw data at the retention time of each peak identified by CODA for the presence of signals with m/z values obtained by subtracting the mass of sodium and adding one (positive ionization) or subtracting the mass of acetyl radical and one (negative ionziation). After peak alignment, signals occurring in controls (ethyl acetate extracts of uninoculated medium) and signals detected in fewer than three of four replicates were discarded. Signals obtained after positive and negative are presented separately because we did not have enough information to decide which pairs of signals originated from the same chemical entities. Normalization of signal intensities was performed as described [40]; furthermore, peak areas for single cultures were divided by fungal biomass and peak areas for dual cultures by the biomass of the producer of the pertinent metabolite, which was identified by comparison with metabolic profiles of single species. Signals of unknown origin, which occurred only in dual cultures but not in single cultures, were normalized by dividing peak areas with the average biomass of both fungi. After normalization, corresponding signals in single and dual cultures were compared. Suppressed metabolites were defined as metabolites whose normalized signal intensities were at least 10-times lower in dual cultures than in single cultures. Induced metabolites were defined as metabolites whose signal intensities were at least 10-times larger in dual cultures than in single cultures. Interaction-specific metabolites were defined as signals found only in dual cultures. In the biotransformation experiment, metabolites extracted from culture supernatant of fungus A were incubated with washed mycelium of fungus B. HPLC-MS profiles of metabolites from fungus A before and after incubation were compared with the profiles of mycelium B incubated in pure medium.

### Qualitative data analysis and descriptive statistics

The HPLC-MS profiles of all samples in form of peak tables were combined in a single matrix containing retention times, m/z values, and peak areas. The matrix was transformed into a binary matrix showing the presence or absence of signals. The matrix was used to study the effect of fungal interactions and cultivation time on the diversity of secreted metabolites and to group samples by cluster analysis (UPGMA clustering using Jaccard’s similarity indices).

### Ethics approval and consent to participate

Not applicable.

### Availability of data and materials

All data and fungal strains used are available.

## Additional file

Additional file 1: Table S1. Species-specific fungal biomass in dual cultures of *Aspergillus niger* (A.n.), *Fusarium verticillioides* (F.v.), and *Clonostachys rosea* (C.r.) (DOCX 28 kb)

## Abbreviation

*A.n.*: *Aspergillus niger*
*C.r.*: *Clonostachys rosea*
*F.v.*: *Fusarium verticillioides*
FB1: fumonisin B1
FB2: fumonisin B2
FB3: fumonisin B3
