# Supplementary material for: Interactions among filamentous fungi Aspergillus niger, Fusarium verticillioides and Clonostachys rosea: fungal biomass, diversity of secreted metabolites and fumonisin production
Source: BMC Microbiol. 2016 May 10;16:83. doi: 10.1186/s12866-016-0698-3 (PMC4862089; doi:10.1186/s12866-016-0698-3)
Supplement: Additional file 1: Table S1. — Species-specific fungal biomass in dual cultures of Aspergillus niger (A.n.), Fusarium verticillioides (F.v.), and Clonostachys rosea (C.r.) (DOCX 28 kb) [file 12866_2016_698_MOESM1_ESM.docx]

**Supporting Information**

**Table S1. Species-specific fungal biomass in dual cultures of *Aspergillus niger* (A.n.), *Fusarium verticillioides* (F.v.), and *Clonostachys rosea* (C.r.)**

Fungal cultures inoculated by conidia at 10^4^ spores/ml were grown in stationary liquid cultures in GM7 medium at 21°C in the dark. Total biomass of the mycelium was determined by weighing. Contribution of each species to the total biomass of dual cultures was determined by co-amplification of a segment of the gene encoding 28S ribosomal RNA, digestions with restriction enzymes and densitometric quantification of species-specific fragments.

| Time (days) | Interacting fungi | Biomass of fungal mycelium in dual cultures ± standard deviation (mg) | | |
| --- | --- | --- | --- | --- |
|  |  | *A.n.* | *F.v.* | *C.r.* |
| 10 | A.n. + F.v | 26 ± 6 | 20 ± 4 | - |
|  | A.n. + C.r. | 31 ± 5 | - | 27 ± 3 |
|  | F.v. + C.r. | - | 43 ± 5 | 21 ± 4 |
| 20 | A.n. + F.v | 107 ± 14 | 14 ± 3 | - |
|  | A.n. + C.r. | 87 ± 6 | - | 53 ± 9 |
|  | F.v. + C.r. | - | 23 ± 7 | 118 ± 6 |
| 30 | A.n. + F.v | 56 ± 5 | 37 ± 7 | - |
|  | A.n. + C.r. | 105 ± 9 | - | 59 ± 2 |
|  | F.v. + C.r. | - | 7 ± 2 | 152 ± 10 |
